# Synthesis and Evaluation of Carbon Black-Containing Hydrogels for the Adsorption of 5-Fluorouracil

**DOI:** 10.3390/gels11110919

**Published:** 2025-11-18

**Authors:** Ritsuko Sekiguchi-Arai, Yoshiko Yamazaki, Takao Sato, Naoki Noma, Kengo Oka, Mitsunobu Iwasaki

**Affiliations:** 1 Graduate School of Science and Engineering, Kindai University, 3-4-1 Kowakae, Higashiosaka, Osaka 5778502, Japan; 2 Medical Materials Laboratory, SEED Co., Ltd., 1030-7 Fukuro, Konosu, Saitama 3690131, Japan; yoshiko_yamazaki@seed.co.jp (Y.Y.); takao_sato@seed.co.jp (T.S.); 3 Division of Joint Research Center, Kindai University, 3-4-1 Kowakae, Higashiosaka, Osaka 5778502, Japan; noma@apch.kindai.ac.jp; 4 Department of Applied Chemistry, Kindai University, 3-4-1 Kowakae, Higashiosaka, Osaka 5778502, Japan; koka@apch.kindai.ac.jp

**Keywords:** 5-fluorouracil, 2-hydroxyethyl methacrylate, carbon black, contact lens, hydrogel, adsorption

## Abstract

The widely used anticancer drug 5-Fluorouracil (5-FU) may potentially elicit adverse side effects on the eyes. To address this problem, we aimed to synthesize hydrogels containing carbon black (CB), a porous material, for use as a 5-FU-adsorbent contact lens material. High-performance liquid chromatography revealed a direct correlation between the specific surface area of CB particles and 5-FU adsorption. CB particles with functional surface groups were characterized by superior dispersibility in monomer solutions, whereas the results of combustion ion chromatography indicated that 5-FU adsorption was higher for hydrogels with a higher water content and that the addition of CB to the hydrogel further enhanced the rate of 5-FU adsorption by 21%. In addition, 5-FU was strongly fixed within the CB-printed hydrogel matrix even after washing, with hydrophobic interactions with CB being established to be highly effective for binding 5-FU. Collectively, the findings of our study revealed that CB-printed hydrogel is a promising novel material for fabricating 5-FU-trapping contact lenses.

## 1. Introduction

**Figure 1:**
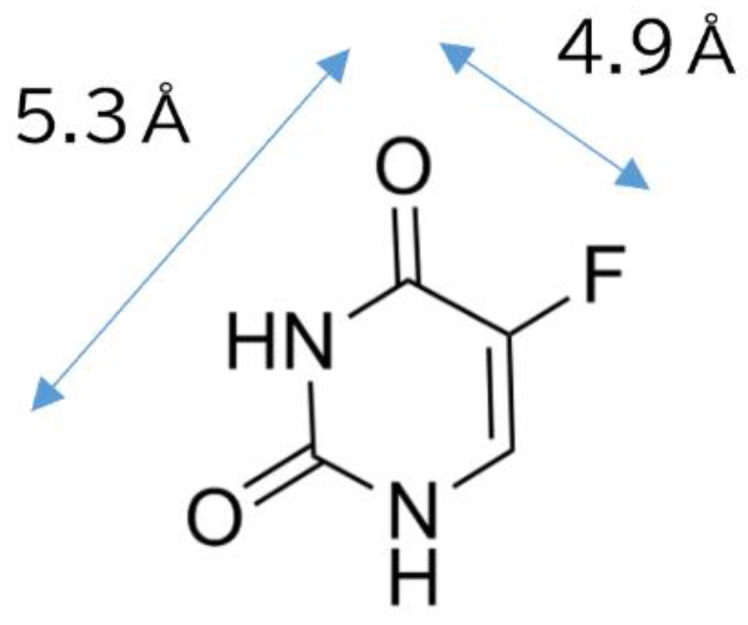
Chemical structure of 5-fluorouracil.

In recent years, there has been a worldwide increase in the incidence of cancer. According to the Global Burden of Disease (GBD) 2023, 18.5 million people were newly diagnosed with cancer, leading to 10.4 million deaths in 2023. This represents a substantial increase from previous estimates. Projections suggest that by 2050, the annual number of new cancer cases will reach 30.5 million and 18.6 million deaths from cancer [1]. However, although the cancer morbidity rates have been increasing, there has also been a corresponding increase in cancer survival rates, primarily attributed to the development of more effective anticancer drugs and treatment methods. There is accordingly a growing need to improve the quality of life after cancer treatment. Current cancer treatments typically involve surgery, radiotherapy, and chemotherapy. Chemotherapy, which utilizes anticancer drugs, is often used in conjunction with surgery and radiotherapy [2]. Anticancer drugs can be classified based on their effects on the cell cycle, with one important class being antimetabolites, which inhibit cancer cell growth by inhibiting DNA synthesis. These drugs imitate DNA components, such as purines and pyrimidines, and have been shown to have enhanced efficacy when combined with other anticancer drugs [3]. For example, tegafur, the first orally administered anticancer drug, is a prodrug of the antimetabolite 5-fluorouracil (5-FU; Figure 1), and TS-1, a combination anticancer drug that contains tegafur, is widely indicated for gastric, colorectal, and pancreatic cancers [4,5].

**Figure 2:**
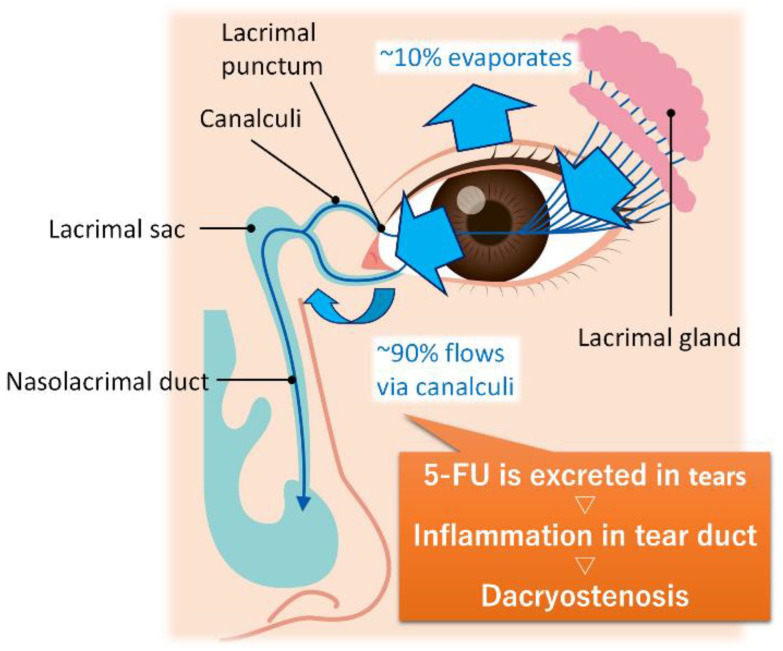
The proposed mechanisms of 5-fluorouracil-induced dacryostenosis.

Similar to other anticancer drugs, TS-1 has been reported to cause a range of potential side effects, including myelosuppression, fatigue, nausea, and anorexia [6]. It has also been found that dispersal of 5-FU to the lacrimal fluid in ocular tissues can lead to side effects in the eyes, including lacrimal obstruction, corneal damage and dry eye disease [5,6,7,8] (Figure 2). Additionally, cases of corneal epitheliopathy have been reported in patients treated with topical 5-FU for ocular surface squamous neoplasia [9]. As a tentative treatment to rectify this problem, although 5-FU is removed from the lacrimal fluid by regularly instilling saline, ocular rinsing may not be sufficiently effective due to the continuous transfer of 5-FU from the blood to the lacrimal fluid through the body’s internal circulation. To address the risk of ocular tissue damage, the continuous removal of 5-FU from the eyes should be considered, including the development of hydrogels that incorporate an effective 5-FU adsorbent for use as a contact lens material.

To date, studies on drug delivery systems have investigated materials that can interact with 5-FU, including hydrogels [10,11], inorganic nanocomposites [12], carbon nanotubes [13], magnetic organic-modified montmorillonite [14], zeolite [15,16], and mesoporous silica [17,18]. Although there have been numerous studies on the release of these substances into the body, there is limited knowledge about 5-FU immobilization. Thus, in this study, we focused on carbon black (CB), an amorphous carbon commonly used as an adsorbent which has a porous structure and high surface area that facilitate the adsorption of a range of different substances, including 5-FU [19,20,21]. For practical applications, it is initially necessary to process CB into shapes that are appropriate for the intended use. The aim of this study was to incorporate CB into a hydrogel to enable convenient molding while still retaining its 5-FU-adsorptive capacity. The use of hydrogels is particularly practical, as the resulting material can be used to fabricate contact lenses. Research on hydrogel materials for contact lenses is currently underway [22,23,24], and in this regard, specifications for vision correction require surface characteristics that interact well with lacrimal fluid, moderate strength, and transparency. However, few studies have reported devices that utilize functionalized fine particles, such as intraocular placement devices. For example, pH-sensitive Eudragit nanoparticle-laden contact lenses have been developed for sustained levobunolol release in glaucoma therapy; however, aggregation of the nanoparticles resulted in reduced lens transparency and wearing comfort [25]. 

According to safety data obtained from the Scientific Committee on Consumer Safety, no ocular irritation has been observed after 24, 48, and 72 h of irrigating rabbit eyes with a solution containing CB [26]. Furthermore, research has been conducted on CB-dispersed colored contact lenses with the aim of reducing photophobia in patients with cystinosis [27], and it has also been found that there are no significant difference in the oxygen permeability of transparent and colored contact lenses (CB-printed hydrogel), thus indicating that the dispersion of CB in contact lenses has no appreciable effects on oxygen permeability [28].

In this study, we fabricated CB-printed hydrogels and evaluated their potential utility as a 5-FU adsorbent, as well as their retention of 5-FU following adsorption. We anticipate that such CB-printed hydrogel composites will find future applications in medical devices, particularly contact lenses.

## 2. Results and Discussion

### 2.1. Characterization of CB Particles

#### 2.1.1. Relationships Among Particle Size, pH, Surface Area, and 5-FU Loading

The 5-FU adsorption and other properties of CB particles are shown in Table 1 and Figure S1. The results indicated a reduction in 5-FU loading with an increase in mean particle size, whereas little correlation was observed between 5-FU adsorption and pH.

**Figure 3:**
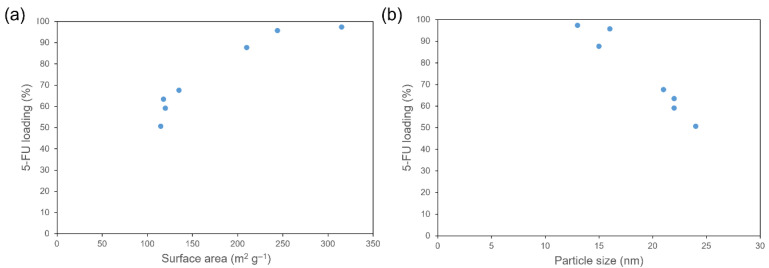
Relationships between 5-FU loading and the specific surface area (**a**) and mean particle size (**b**) of carbon black.

The surface area of CB was found to be directly correlated with the amount of 5-FU loaded (Figure 3a). For example, the 5-FU loading of CB2 (surface area: 315 m^2^ g^−1^) was 46.7% higher than that of CB1 (surface area: 115 m^2^ g^−1^). In addition, we detected a negative association between 5-FU loading and mean CB particle size (Figure 3b). These results indicate that CB effectively fixed 5-FU via hydrophobic interactions between CB and 5-FU [29].

#### 2.1.2. Dispersion of Different CBs in the Hydrogel Monomer

CB–hydrogel composites were produced using different CBs. The dispersibility of CB in the HEMA monomer, the raw material of hydrogels, is strongly dependent on the polarity and viscosity of the monomer. The polymerization reaction should not be inhibited by CB particles and should occur in the monomer solution with well-dispersed CB particles. In this regard, we found that CB2, which was characterized by the highest 5-FU adsorption, had poor dispersibility in the monomer and, consequently, no homogeneous hydrogel was obtained from either the monomer or CB2. In contrast, homogeneous hydrogels with dispersed CB were obtained using CB1 and CB6, whereas CB2 was comparatively less dispersed in the monomer solution. CB has characteristic functional groups on its surface, and when CB particles are suspended in water, the pH of the aqueous solution is altered accordingly [30]. Aqueous solutions of CB1 and CB6 were found to be acidic, with pH values of 3 and 5, respectively, indicating the presence of carboxyl and/or hydroxyl groups on the surfaces of these CBs [31,32]. These surface functional groups interact with the polar HEMA monomer to form a well-dispersed CB solution. In the presence of CB, polymerization of the monomers proceeds smoothly via a radical polymerization reaction. Although we found that pH had little effect on 5-FU adsorption, it did, nevertheless, influence the dispersibility of CB in the monomer solution and the formation of homogeneous hydrogels. Thus, although CB2 was characterized by the highest 5-FU adsorption in solution, its lack of functional groups precluded an efficient dispersal in the hydrogel matrix. Accordingly, subsequent evaluations of the physicochemical properties of hydrogels were based on analyses of CB1 and CB6, as these produced homogeneous CB-dispersed hydrogels.

#### 2.1.3. Particle Size

**Figure 4:**
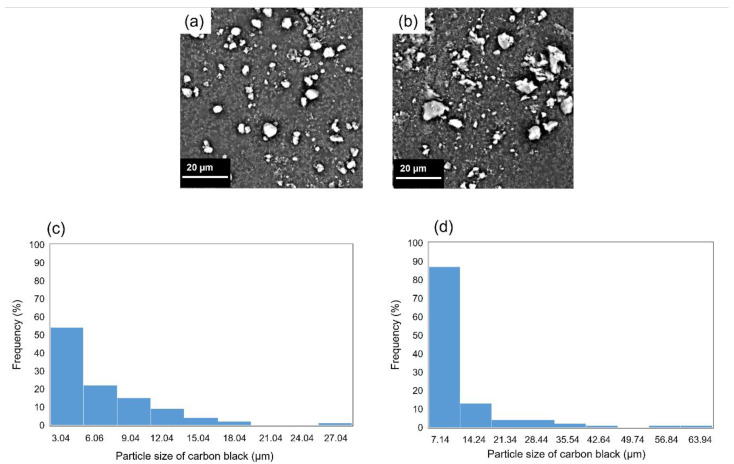
Scanning electron micrographs of (**a**) CB1 and (**b**) CB6 particles. Histograms of the particle sizes of (**c**) CB1 and (**d**) CB6.

**Figure 5:**
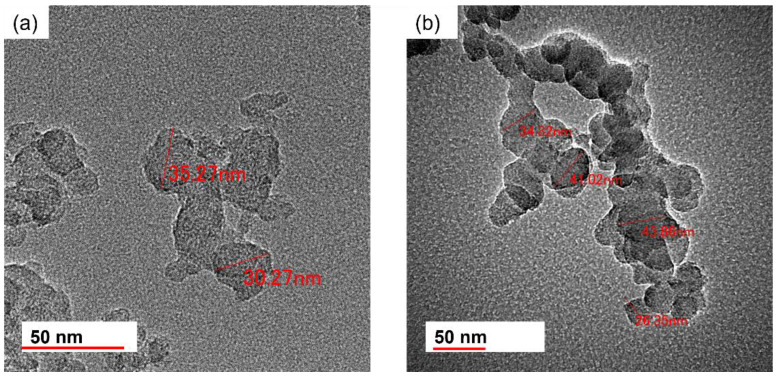
Transmission electron micrographs of (**a**) CB1 and (**b**) CB6 particles.

Scanning and transmission electron micrographs of CB1 and CB6 particles are shown in Figure 4 and Figure 5, respectively. The size of the CB particles generally fell within the range between 25 and 45 nm and tended to be characterized by substantial agglomeration. SEM observations revealed that the mean secondary particle size was approximately 0.98 µm and 0.97 µm for CB1 and CB6, respectively.

#### 2.1.4. Specific Surface Area and Pore Size

**Figure 6:**
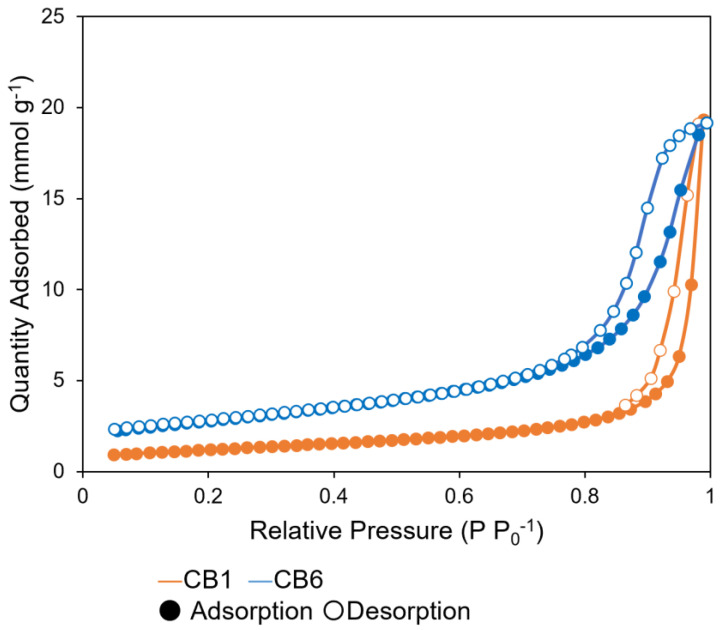
Nitrogen adsorption–desorption isotherms for carbon black.

The nitrogen adsorption–desorption isotherms of CB1 and CB6 are shown in Figure 6. According to the IUPAC classification, each sample was characterized by a type-V isotherm [33]. There was a steep increase in adsorption at a P/P_0_ ratio of approximately 0.8, indicating the monolayer adsorption of nitrogen on the microporous channels of CB. Subsequently, multilayer adsorption was observed. The specific surface areas and pore sizes were estimated using the Brunauer–Emmett–Teller and Barrett–Joyner–Halenda methods, respectively (Table 2). Whereas CB1 and CB6 were found to have similar pore volumes, the particles of CB6 had a larger specific surface area and smaller pore size than those of CB1. Consequently, compared with CB1, CB6 was observed to adsorb larger amounts of 5-FU (Table 2). Thus, the larger specific surface area and smaller pore size of CB6 resulted in a higher adsorption of 5-FU.

### 2.2. Characterization of CB-Containing Hydrogels

#### 2.2.1. Effects of the CB Incorporation Method

**Figure 7:**
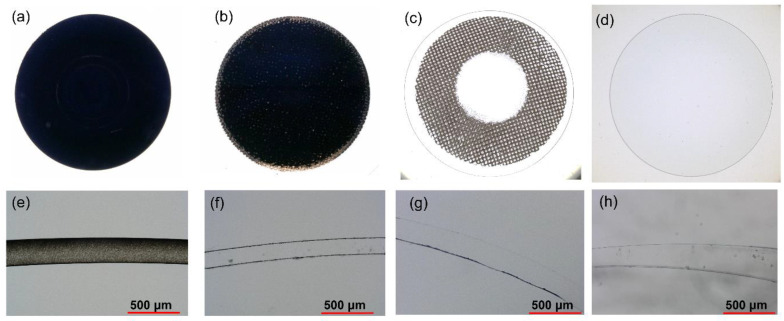
Photomicrographs of (**a**) CB-dispersed, (**b**) CB-printed, (**c**) CB-printed with a transparent optic zone, and (**d**) transparent hydrogels. Cross-sectional images of (**e**) CB-dispersed, (**f**) CB-printed, (**g**) CB-printed with a transparent optic zone, and (**h**) transparent hydrogels. All hydrogels comprised HG56 and were 14 mm in diameter. CB hydrogels contained 1 wt% CB1. Scale bars = 500 µm.

Figure 7a–d shows digital micrographs of CB-dispersed, CB-printed, CB-printed with a transparent optic zone, and transparent hydrogels, respectively. The CB coverage (black portions) in the hydrogel lenses was 99.5% for the CB-dispersed hydrogel (Figure 7a) and 96.1% for the CB-printed hydrogel (Figure 7b), thus indicating that in both these hydrogels, the CB particles were well dispersed. Cross-sectional micrographs of the hydrogels are shown in Figure 7e–h for CB-dispersed, CB-printed, CB-printed with a transparent optic zone, and transparent hydrogels, respectively. In Figure 7e, almost all parts of the cross-section of the CB-dispersed hydrogel are black, indicating that the CB particles are very well dispersed in the monomer solution, and that the monomers had uniformly polymerized to form the CB1-dispersed hydrogel. Similarly, CB particles were uniformly coated on the CB-printed hydrogel surface. Comparable results were obtained for CB6.

**Figure 8:**
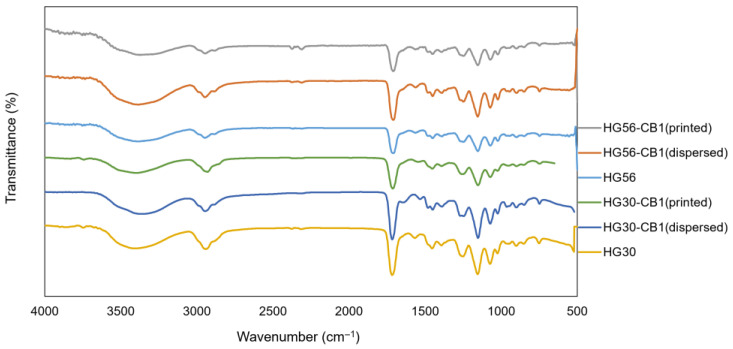
FTIR spectra of HG30 and HG56 hydrogels without CB, with CB-dispersed, and with CB-printed.

The chemical structure of the hydrogel was characterized by FTIR spectroscopy (Figure 8). The FTIR spectrum of HG30 exhibited vibrational bands in the hydroxyl (O–H) (3371 cm^−1^) and carbonyl (C=O) (1718 cm^−1^) stretching regions, and bands 1451 cm^−1^ and 2928 cm^−1^ associated with the symmetric and asymmetric C–H stretching vibrations (CH_2_ and CH_3_ groups), respectively. The results indicated that similar surface characteristics were obtained regardless of the presence or absence of MAA and CB, as all samples exhibited identical peak patterns.

**Figure 9:**
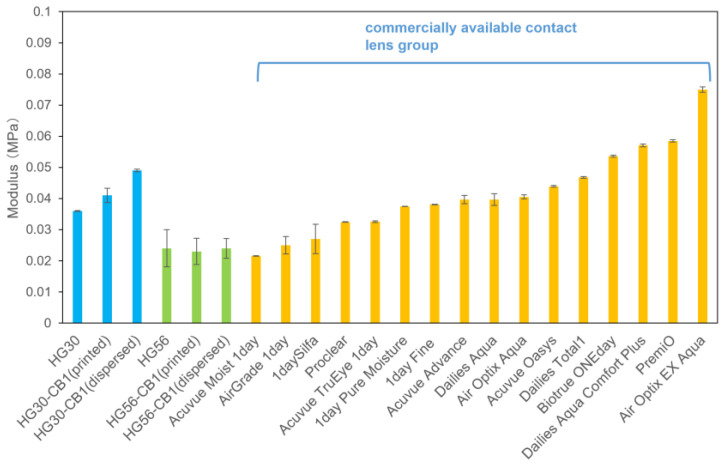
Storage moduli at a shear strain of 0.15% for HG30 and HG56 contact lenses compared with selected commercially available contact lenses. Data are presented in mean ± standard deviation, *n* = 3.

Figure 9 shows the results of the rheological measurements. For the no CB, CB-dispersed, and CB-printed HG30 hydrogels, we obtained values of 0.036, 0.041, and 0.049 MPa, respectively, for the storage moduli at a shear strain of 0.15%, with corresponding values of 0.024, 0.023, and 0.024 MPa for the no CB, CB-dispersed, and CB-printed HG56 hydrogels. Notably, the rheological values obtained for the sample groups were found to be comparable to those obtained for the commercial lens group, indicating their suitability for use as contact lenses.

**Figure 10:**
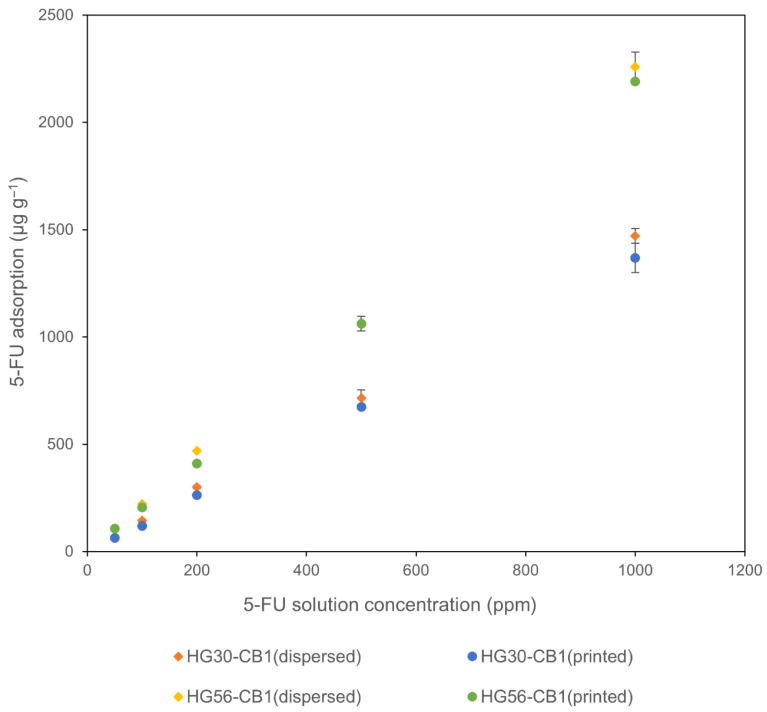
Relationship between the 5-FU solution concentration and 5-FU loading in four hydrogels. Data are presented in mean ± standard deviation, *n* = 3.

Interactions between CB and 5-FU were investigated in different CB-containing hydrogels. These hydrogels were immersed in aqueous solutions containing differing concentrations of 5-FU, and we determined the association between 5-FU adsorption and the concentration of the 5-FU solution (Figure 10). For each of the four assessed hydrogels, the amount of 5-FU adsorbed was found to be directly proportional to the concentration of the 5-FU solution. Thus indicating that 5-FU was effectively adsorbed onto the hydrogels. Moreover, we established that compared with HG30, in each of the different 5-FU solutions, the HG56 hydrogel adsorbed a greater amount of 5-FU, which can be attributed to the higher water content of this hydrogel.

A number of previous studies have focused on drug delivery systems and the adsorption and release of 5-FU at high concentrations [10,15,17,34], and the adsorbents zeolites [10,15,16], mesoporous silica [17], and chitosan [34] have been identified as effective in the adsorption of 5-FU. Accordingly, these materials have been used in drug delivery systems that facilitate the gradual release of 5-FU from the adsorbents. In contrast, in the present study, we focused on the fixation of 5-FU to CB, with the objective of preventing its subsequent release. In this regard, the ideal adsorbent should have the capacity to adsorb as much 5-FU as possible without releasing it. Thus, we examined the adsorption of 5-FU in solution by CB2 at 5-FU concentrations of less than 1000 ppm, and accordingly found that CB performs satisfactorily as an adsorbent. Given that previous studies have generally examined 5-FU adsorption at high 5-FU concentrations (e.g., 1%), the results of these studies cannot be directly compared with the results reported herein [10,15].

When HG30 and HG56 are immersed in a 500 ppm 5-FU solution, the calculated amounts of adsorbed 5-FU (300 and 560 µg g^−1^, respectively) were found to be considerably lower than the measured values (650 and 1100 µg g^−1^, respectively), a trend that was consistent with our observations at the other 5-FU concentrations (Figure 10). In this context, Parisi et al. have suggested that the hydrophilic nature of 5-FU, combined with its aromaticity and capacity to form hydrogen bonds, may facilitate its retention in the hydrogel matrix [35]. CB can also sufficiently adsorb 5-FU, and thus the significant increase observed in the adsorption of 5-FU can be attributed to the combined contribution of both hydrogel matrix and CB.

Additionally, there was no significant difference between the CB-dispersed and CB-printed hydrogels with respect to 5-FU adsorption, indicating that the CB incorporation method had no detectable adverse influence on the adsorption of 5-FU. However, in subsequent experiments, we used a CB-printed hydrogel, as its preparation is simpler than that of the CB-dispersed hydrogel.

#### 2.2.2. Adsorption of CB1 and CB6 Hydrogels Compared with Transparent Hydrogels

**Figure 11:**
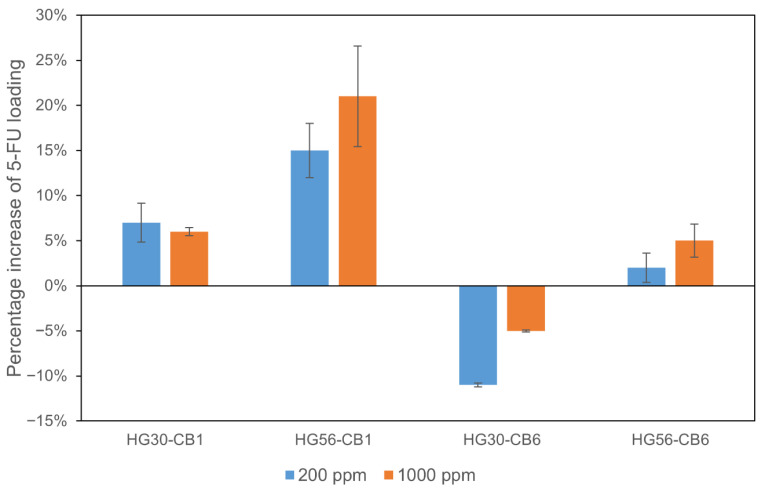
Percentage increase in 5-FU loading in CB-printed hydrogels compared with that in transparent hydrogels. Data are presented in mean ± standard deviation, Significant differences (*p* < 0.05, *n* = 3) were observed between HG30-CB1 and HG56-CB1, and between HG30-CB6 and HG56-CB6.

Having immersed different hydrogels in aqueous solutions containing 200 and 1000 ppm 5-FU, we proceeded to evaluate the amounts of 5-FU adsorbed on these hydrogels (Figure 11). For HG30-CB1, regardless of the 5-FU concentration, we recorded similar 5-FU adsorption capacities. However, the amounts of 5-FU adsorbed by HG56-CB1 were found to be significantly higher than those adsorbed by HG30-CB1.

Unlike CB1 hydrogels, CB6-printed hydrogels showed no significant increase in 5-FU adsorption with an increase in concentration of the 5-FU solution. This can be attributed to the relatively larger pores of CB1 (Table 2), which would be more conducive to accommodating the polymer chains. Consequently, 5-FU was more readily adsorbed on CB1. which probably adsorbed more 5-FU than did CB6 in HG30 and HG56.

The less pronounced adsorption of 5-FU by HG30-CB6 compared with that of HG30, indicates that the presence of HEMA polymer chains may have inhibited the adsorption of 5-FU onto CB6. However, compared with HG56, we observed 2% and 5% increases in the adsorption of 5-FU by HG56-CB6 at 200 and 1000 ppm, respectively, which can be ascribed to the higher water content of HG56 that contributed to reducing the density of the polymer. Furthermore, at both assessed 5-FU concentrations (200 and 1000 ppm), the CB1-printed hydrogels [HG30-CB1(1%) and HG56-CB1(1%)] were found to adsorb larger amounts of 5-FU than the transparent hydrogels.

The 5-FU adsorption properties of CB-printed hydrogels were found to be highly dependent on the type of CB used. In particular, hydrogel printed with CB1 with a large pore size (HG30-CB1) adsorbed larger amounts of 5-FU than the hydrogel alone (HG30). Notably, even though one surface of the hydrogel was covered with CB1, the amounts of 5-FU adsorbed increased. In contrast, compared with that of HG30, the less pronounced adsorption of 5-FU by HG30-CB6 would tend to indicate that the presence of polymer chains hinders the adsorption of 5-FU by CB6 in hydrogels. This finding indicates that the polymer chains may effectively occlude the smaller pores of CB6, thereby impeding the adsorption of 5-FU. Comparatively, HG56-CB6, which had a higher water content and lower polymer density, was characterized by a higher 5-FU adsorption capacity. Notably, 5-FU is present in the lacrimal fluid at concentrations of less than a few hundred ppm [36], and thus, HG56-CB1, which facilitates good uptake at low concentrations of 5-FU, may have potential application for 5-FU fixation in ocular tissues.

#### 2.2.3. Evaluation of Adsorption Performance

**Figure 12:**
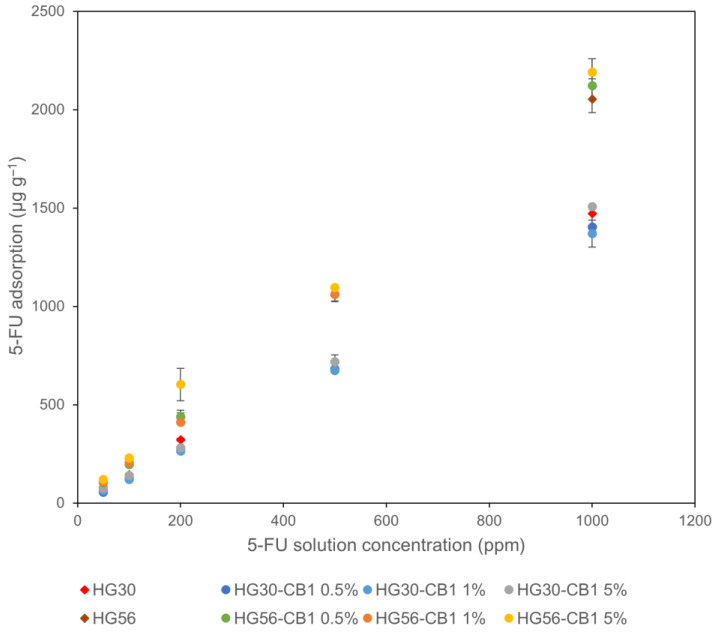
Relationship between the 5-FU solution concentration and 5-FU loading in different hydrogels. Data are presented in mean ± standard deviation, *n* = 3.

Figure 12 shows the adsorption of 5-FU on CB1-printed hydrogels (HG30-CB1 and HG56-CB1) following immersion in a 5-FU solution, and indicates that for all assessed hydrogels, 5-FU uptake increases in proportion to the concentration of 5-FU.

The enhanced adsorption of 5-FU by CB-containing hydrogels is assumed to be primarily attributable to three factors, namely, the hydrophobic interactions between CB and 5-FU [24,37], interactions with the polymer chains forming the hydrogel [35], and solubility and diffusion into the hydrogel due to its water content [19]. Furthermore, with an increase in the CB1 content from 1% to 5%, an increase in the amount of 5-FU adsorbed was observed for HG30-CB1 and HG56-CB1. The hydrophobic interactions between 5-FU and CB1 at concentrations of up to 5% CB1 were found to be conducive to the effective adsorption of the former on the latter [24,37].

Table 3 shows the 5-FU adsorption per unit weight of hydrogel and CB1-printed hydrogel (5%) after soaking in a 1000 ppm 5-FU solution. The amount of 5-FU adsorbed per unit weight of CB1 was found to be 147% and 233% higher than that of hydrogels (HG30 and HG56) for HG30-CB (5%) and HG56-CB (5%), respectively, thereby indicating the notably superior adsorptive capacity CB1 compared with that of the transparent hydrogels.

#### 2.2.4. Evaluation of 5-FU Fixation After Washing

To investigate the fixation of 5-FU in the hydrogels, the transparent and CB1-printed hydrogels were immersed in a 5-FU solution and thereafter washed with PBS. The residual amounts of 5-FU remaining in the hydrogels after washing are shown in Table 4. The amount of adsorbed 5-FU per unit weight in HG30 and HG30-CB1 (printed) hydrogels was 294 µg g^−1^ and 315 µg g^−1^, respectively, which compared with the data presented in Figure 12, indicates that in both cases, the adsorption of 5-FU was less pronounced. A comparison of the amounts adsorbed before and after washing, revealed that between 89% and 95% of the adsorbed 5-FU had been retained in CB1-printed hydrogels, compared with the 76–91% retained in hydrogels lacking CB1, thereby highlighting the efficacy of CB1 in capturing 5-FU.

Compared with the transparent hydrogels (HG30 and HG56), the CB1-printed gels (HG30-CB1 and HG56-CB1) showed increases of 7% and 15%, respectively, in the adsorption of 5-FU following immersion in a 200 ppm 5-FU solution. When immersed in a 5-FU solution at the higher concentration of 1000 ppm, the CB-printed gels (HG30-CB1 and HG56-CB1) showed an increase in 5-FU adsorption of 6% and 21%, respectively, compared with the transparent hydrogels (HG30 and HG56), thus indicating the particular efficacy of HG56-CB1 at higher 5-FU concentrations.

Hydrophobic interactions between 5-FU and CB [24,37] and 5-FU and the hydrogel [35] have been established to be resistant to disruption following water rinsing. Notably, the magnitude of hydrophobic interactions has been found to be greater in interactions between 5-FU and the hydrogel, and, previously, post-adsorption 5-FU releases of 80% and 39–55% have been reported for mesoporous silica [17] and chitosan nanoparticles [34], respectively. Consequently, with retentions of between 89% and 95%, even after washing, CB-containing hydrogels can be considered particularly effective adsorption media.

Despite our important findings, this study has certain limitations. We did not perform any in vitro or in vivo experiments using CB-printed hydrogels as a contact lens material. In the future, the cytocompatibility of CB-printed hydrogels and their adsorption of 5-FU from lacrimal fluid should accordingly be evaluated.

## 3. Conclusions

In this study, we demonstrated that CB is an effective material for capturing 5-FU, with a strong adsorptive capacity mediated via hydrophobic interactions. In particular, we established that CB containing carboxyl or hydroxyl groups had optimal desirable properties and was well dispersed within hydrogels. CB-dispersed hydrogels exhibit overall black coloration, presenting a drawback for contact lens use. In contrast, CB-printed hydrogels retain transparency in the optical zone, demonstrating their feasibility for contact lens applications. The incorporation of CB into the hydrogel matrix was found to enhance the adsorption of 5-FU, with the capacity of CB-printed hydrogels being approximately double that of the unprinted hydrogels. Furthermore, we found that a substantial proportion of the adsorbed 5-FU remained bound to the CB-printed hydrogel even after thorough washing. The findings of this study indicate that CB-printed hydrogels possess excellent 5-FU adsorption and retention capabilities, suggesting their potential as efficient materials for 5-FU capture. In addition, even CB-dispersed hydrogels can be readily modified, thereby highlighting their versatility for future applications. Future studies will aim to elucidate the adsorption characteristics of 5-FU in greater detail, taking into account the structural properties of the gels.

## 4. Materials and Methods

### 4.1. Materials

5-FU was purchased from Tokyo Chemical Industry (Tokyo, Japan), and CB was obtained from the following manufacturers: MA7 (CB1) from Mitsubishi Chemical Corporation (MCC; Tokyo, Japan); SB935 (CB2), SB905 (CB3), SUNBLACK25 (CB4), and SUNBLACK45 (CB5) from Asahi Carbon (Niigata, Japan); and #8300/F (CB6) and #7550SB/F (CB7) from Tokai Carbon (Tokyo, Japan). Seven commercially available CB samples were selected based on comparisons of pore size, specific surface area, and the pH of their aqueous dispersions, using CB1—previously applied in color contact lenses—as the reference.

2-Hydroxyethyl methacrylate (HEMA) was purchased from Mitsubishi Gas Chemical Company (Tokyo, Japan), trimethylolpropane trimethacrylate (TMPTMA) from Kyoeisha Chemical (Osaka, Japan), ethylene glycol dimethacrylate (EGDMA) from MCC, methacrylic acid (MAA) and 2,2′-azobis(isobutyronitrile) (AIBN) from FUJIFILM Wako Pure Chemical (Osaka, Japan), and poly-2-hydroxyethyl methacrylate (pHEMA) from Asahi Kasei Finechem (Osaka, Japan).

The following contact lenses were purchased for rheological comparison: SEED 1dayPure moisture, SEED AirGrade 1day UV W-Moisture, SEED 1daySilfa, and 1day fine UV from SEED (Tokyo, Japan); Proclear 1day from Cooper Vision Japan (Tokyo, Japan); Biotrue ONEday from Bausch & Lomb Japan (Tokyo, Japan); Acuvue Moist 1day, Acuvue TruEye 1day, Acuvue Oasys, and Acuvue Advance from Johnson & Johnson Vision Care (Tokyo, Japana); Dailies Aqua, Air Optix Aqua, Air Optix EX Aqua, Dailies Total1, and Dailies Aqua Comfort Plus from Alcon Japan (Tokyo, Japan); and PremiO from Menicon (Nagoya, Aichi, Japan).

### 4.2. Hydrogel Synthesis

#### 4.2.1. CB-Dispersed Hydrogel

Hydrogels with differing aqueous contents were synthesized using different monomer mixtures. HG30 (30% water content) was synthesized using a mixture of 99.5 wt% HEMA and 0.5 wt% EGDMA, whereas HG56 (56% water content) was synthesized using a mixture of 96 wt% HEMA, 3.3 wt% MAA, 0.1 wt% EGDMA, and 0.6 wt% TMPTMA. Both monomer mixtures were initiated with AIBN (3000 ppm). CB (1–5 wt%) was then added to the monomer mixtures, and the resulting solutions were mixed for 3 h at room temperature prior to being poured into disk-shaped polypropylene (PP) molds. Following polymerization at 100 °C for 12 h, the cured polymers (HG30 and HG56) were removed from the molds and soaked in phosphate-buffered saline (PBS) for 3 h to hydrate. Subsequently, the CB-dispersed HG30 and HG56 hydrogels were sterilized by autoclaving at 121 °C for 20 min in accordance with the ISO 17665-1 standard [38].

#### 4.2.2. CB-Printed Hydrogel

pHEMA was mixed with HEMA and stirred overnight at room temperature. CB ink was prepared by mixing 20–30% CB (CB1 or CB6) and pHEMA/HEMA to form hydrogels (HG30 and HG56). Furthermore, the CB ink was printed on the PP mold surface using a silicon pad and dried at 90 °C for 10 min. The hydrogels were then prepared using the same procedure as that used to prepare the CB-dispersed hydrogels.

### 4.3. Characterization of Morphology and Adsorption Properties

#### 4.3.1. CB and Hydrogel Morphology

The morphologies of the CB particles were observed using scanning electron microscopy (SEM: SEM Phenom ProX; Thermo Fisher Scientific K.K., Tokyo, Japan) and transmission electron microscopy (TEM: JEM-2100; JEOL Ltd., Tokyo, Japan) with an acceleration voltage of 200 V and electric current of 140 μA). ImageJ software (Java1.8.0-internal version 0.5.8) was used to determine the size of the CB nanoparticles. The surface area and pore morphology of the samples were analyzed via nitrogen adsorption–desorption isotherms using a Micromeritics TriStar 3000 Surface Area Analyzer (Shimadzu Co., Kyoto, Japan) with the following settings: nitrogen gas, initial relative pressure 0.05, final relative pressure 0.995, number of points 50, and progression liner. All samples were initially vacuum-dried at 100 °C for 1 h to remove any moisture and adsorbed gases. The morphology and CB coverage of the hydrogels was observed using a VHX6000 digital microscope (Keyence Corporation, Osaka, Japan).

Having initially determined the weights of the wet hydrogel and dried hydrogel following evaporation by heating at 120 °C for 2 h, the water content of the hydrogels was calculated using Equation (1) as follows:

(1) $$Water\;content\;\left(\%\right)=\frac{W_{w}-W_{d}}{W_{w}}\times 100\%,$$

*W_w_*: weight of wet hydrogel, *W_d_*: weight of dried hydrogel

The structures for the synthesized hydrogels were characterized by Fourier transform infrared spectra (FTIR: FT/IR-4100, JASCO Corporation, Tokyo, Japan) at room temperature with 32 scans spanning a spectral range of 4000–500 cm^−1^ and a resolution of 2.0 cm^−1^. The hydrogels were characterized directly by attenuated total reflection (ATR) mode. Rheological measurements were performed using a rheometer (Modular Compact Rheometer MCR302; Anton Paar, Japan K.K., Tokyo, Japan) with the following settings: PP08/S parallel plate, with a sand-blasted plate cap, normal force = 0.3 N, frequency = 10 rad s^−1^, and shear strain = 0.01–100%. The sample holder was maintained at 25 °C.

#### 4.3.2. Measurement of 5-FU Adsorption onto CB Particles

5-FU was dissolved in PBS (200 ppm), with 50 mg of CB powder subsequently being added to 4 mL of the 5-FU solution. The mixture was then shaken for 24 h at room temperature, followed by filtration of the resultant solution. 5-FU concentration was determined using high-performance liquid chromatography (HPLC: LC-2000Plus; JASCO Corporation) under the conditions shown in Table 5. This method was developed and optimized with reference to the study conducted by Nakamoto et al. [39].

5-FU loading was calculated using Equation (2) as follows:

(2) $$Drug\;loading=\frac{\mathrm{Initial}\;\mathrm{conc}.\;\mathrm{of}\;\mathrm{drug}-\mathrm{conc}.\;\mathrm{of}\;\mathrm{drug}\;\mathrm{after}\;\mathrm{soaking}}{\mathrm{Initial}\;\mathrm{conc}.\;\mathrm{of}\;\mathrm{drug}}\times 100\%$$

#### 4.3.3. Measurement of 5-FU Adsorption onto CB-Containing Hydrogels

A 5-FU soaking solution was prepared by dissolving 5-FU at different concentrations (50, 100, 200, 500, and 1000 ppm) in PBS, and hydrogel (25 mg) was added to 4 mL of this soaking solution. The solution was shaken at room temperature for 24 h, with the hydrogels being withdrawn from the solution at certain time intervals, followed by wiping and subsequent drying at 90 °C for 4 h. 5-FU adsorption was measured based on the fluorine content of the dried hydrogels using combustion ion chromatography (HSU-35/SQ-5/SQ-10; Yanaco LID Co., Ltd., Kyoto, Japan and Dionex ICS-2100, Thermo Fisher Scientific K.K.), and 5-FU loading was calculated using Equation (3) as follows:

(3) $$Drug\;loading({\mu g\;g}^{-1})=\frac{\mathrm{Fluorine}\;\mathrm{content}({\mu g\;g}^{-1})}{18.998\;\left(\mathrm{MW}\;\mathrm{of}\;F\right)}\times 130.078\;(\mathrm{MW}\;\mathrm{of}\;\text{5-FU})$$

A 5-FU adsorption per unit weight of hydrogel after soaking in a 5-FU solution. which was calculated using Equation (4) as follows.

(4) $$5-FU\;adsorption\;per\;unit\;weight\;\left({\mu g\;g}^{-1}\right)=\frac{A_{ch}-A_{hy}}{W_{CB}},$$

*W_CB_*: weight of CB, A_ch_: amount of 5-FU adsorbed onto the CB-printed hydrogel,

*A_hy_*: amount of 5-FU adsorbed onto the hydrogel.

### 4.4. Statistical Analysis

The reported values are expressed as the arithmetic mean and standard deviation of the average of at least three samples for each condition. One-way analysis of variance was performed to compare differences between the groups. Data were considered statistically significant at *p* < 0.05.

## Figures and Tables

**Table 1:** Mean particle size, specific surface area, pH, and 5-FU loading of different carbon blacks.

Carbon Black	Mean Particle Size ^(a)^	Specific Surface Area ^(b)^	pH ^(c)^	5-FU Loading
	(nm)	(m^2^ g^−1^)		(%)
CB1	24	115	3	50.7
CB2	13	315	7.5	97.4
CB3	15	210	7.5	87.7
CB4	22	118	3	63.4
CB5	22	120	3	59.2
CB6	16	244	5	95.7
CB7	21	135	7.5	67.6

(a) Mean diameter determined via electron microscopy. (b) Specific surface area calculated using the Brunauer–Emmett–Teller equation based on the amount adsorbed nitrogen. (c) pH measured for a mixture of carbon black and distilled water using a glass electrode pH meter.

**Table 2:** Physiochemical properties of CB1 and CB6.

	CB1	CB6
Surface area (m^2^ g^−1^)	101	219
Pore volume (cm^3^ g^−1^)	0.67	0.66
Pore size (Å)	266	123

**Table 3:** Amounts of 5-FU adsorbed per unit weight of hydrogel and CB.

5-FU Adsorbed Per Unit Weight (µg g^−1^)		
	Hydrogel	CB1
HG30	1472 ± 34	2157 ± 34
HG56	2054 ± 68	4792 ± 68

**Table 4:** Amounts of residual 5-FU fixed on four assessed hydrogels after washing with phosphate-buffered saline.

Concentration of 5-FU Solution (ppm)	Residual 5-FU (µg g^−1^)			
	HG30	HG30-CB1	HG56	HG56-CB1
200	294 ± 7	315	342	394 ± 10
1000	1232 ± 14	1301 ± 68	1609 ± 34	1951 ± 34

**Table 5:** High-Performance Liquid Chromatography Conditions.

Column	Spherisorb ODS2 Column 5 µm, 4.6 mm × 250 mm (Waters)
Mobile phase	Methanol/Acetic acid (10 µM) = 10/90
Flow rate	0.6 mL/min
Detection	UV 250 nm
Temperature	40 °C

## Data Availability

The datasets presented in this article are not readily available because the data are being used in an ongoing study. Requests to access the datasets should be directed to the corresponding author.

## Supplementary Materials

The following supporting information can be downloaded at: https://www.mdpi.com/article/10.3390/gels11110919/s1, Figure S1: A high-performance liquid chromatogram for CB1.

## References

[1] GBD 2023 Cancer Collaborators (2025). The global, regional, and national burden of cancer, 1990–2023, with forecasts to 2050: A systematic analysis for the Global Burden of Disease Study 2023. Lancet.

[2] Rich T.A., Shepard R.C., Mosley S.T. (2004). Four decades of continuing innovation with fluorouracil: Current and future approaches to fluorouracil chemoradiation therapy. J. Clin. Oncol..

[3] Kohn K.W., Jackman J., O’Connor P.M. (1994). Cell cycle control and cancer chemotherapy. J. Cell Biochem..

[4] Tabuse H., Kashiwagi H., Hamauchi S., Tsushima T., Todaka A., Yokota T., Machida N., Yamazaki K., Fukutomi A., Onozawa Y. (2016). Excessive watering eyes in gastric cancer patients receiving S-1 chemotherapy. Gastric Cancer.

[5] Yasui H., Kawakami T., Kashiwagi H., Mori K., Omae K., Kasai J., Yoshisue K., Kawahira M., Tsushima T., Machida N. (2019). Pharmacokinetics of S-1 monotherapy in plasma and in tears for gastric cancer patients. Int. J. Clin. Oncol..

[6] Zhuang Z.X., Zhu H., Wang J., Zhu M.G., Wang H., Pu W.Y., Bian H.H., Chen L., Zhang H. (2013). Pharmacokinetic evaluation of novel oral fluorouracil antitumor drug S-1 in Chinese cancer patients. Acta Pharmacol. Sin..

[7] Eiseman A.S., Flanagan J.C., Brooks A.B., Mitchell E., Pemberton C. (2023). Ocular surface, ocular adnexal, and lacrimal complications associated with the use of systemic 5-fluorouracil. Ophthalmic. Plast. Reconstr. Surg..

[8] Vitiello L., Lixi F., Coco G., Giannaccare G. (2024). Ocular Surface Side Effects of Novel Anticancer Drugs. Cancers.

[9] Lan X., Zhang D., Luo S., Fang X., Xie Z., Lin Y., Yang J., Qin X., Shi J., Liu A. (2025). Topical 5-fluorouracil 1% versus surgical removal as a primary treatment modality for ocular surface squamous neoplasia: A comparison of recurrences and side effects. BMC Ophthalmol..

[10] Spatarelu C.P., Chiriac A.L., Cursaru B., Iordache T.V., Gavrila A.M., Cojocaru C.T., Botez R.E., Trica B., Sarbu A., Teodorescu M. (2020). Composite nanogels Based on zeolite-poly(ethylene glycol) diacrylate for controlled drug delivery. Nanomaterials.

[11] Arafat M., Fouladian P., Wignall A., Song Y., Parikh A., Albrecht H., Prestidge C.A., Garg S., Blencowe A. (2020). Development and in vitro evaluation of 5-fluorouracil-eluting stents for the treatment of colorectal cancer and cancer-related obstruction. Pharmaceutics.

[12] Chen S., Hao X., Liang X., Zhang Q., Zhang C., Zhou G., Shen S., Jia G., Zhang J. (2016). Inorganic nanomaterials as carriers for drug delivery. J. Biomed. Nanotechnol..

[13] Kamble R.V., Bhinge S.D., Mohite S.K., Randive D.S., Bhutkar M.A. (2021). In vitro targeting and selective killing of mcf-7 and colo320dm cells by 5-fluorouracil anchored to carboxylated SWCNTs and MWCNTs. J. Mater. Sci. Mater. Med..

[14] Sağir T., Huysal M., Durmus Z., Kurt B.Z., Senel M., Isik S. (2016). Preparation and in vitro evaluation of 5-flourouracil loaded magnetite-zeolite nanocomposite (5-FU-MZNC) for cancer drug delivery applications. Biomed. Pharmacother..

[15] Datt A., Burns E.A., Dhuna N.A., Larsen S.C. (2013). Loading and release of 5-fluorouracil from HY zeolites with varying SiO_2_/Al_2_O_3_ ratios. Micropor. Mesopor. Mater..

[16] Servatan M., Zarrintaj P., Mahmodi G., Kim S.J., Ganjali M.R., Saeb M.R., Mazafari M. (2020). Zeolites in drug delivery: Progress, challenges and opportunities. Drug Discov. Today.

[17] Moodley T., Singh M. (2019). Polymeric mesoporous silica nanoparticles for enhanced delivery of 5-fluorouracil in vitro. Pharmaceutics.

[18] Vallet-Regi M., Colilla M., Izquierdo-Barba I., Manzano M. (2017). Mesoporous silica nanoparticles for drug delivery: Current insights. Molecules.

[19] Hiroaki A. (2016). Manufacturing process and characterization of carbon black. TANSO.

[20] Wang L., Gao J., An Z., Zhao X., Yao H., Zhang M., Tian Q., Zhai X., Liu Y. (2019). Polymer microsphere for water-soluble drug delivery via carbon dot-stabilizing W/O emulsion. J. Mater. Sci..

[21] Nasari M., Semnani D., Hadjianfar M., Amanpour S. (2020). Poly (ε-caprolactone)/poly (N-vinyl-2-pyrrolidone) core–shell nanofibers loaded by multi-walled carbon nanotubes and 5-fluorouracil: An anticancer drug delivery system. J. Mater. Sci..

[22] Jones L., Hui A., Phan C.M., Read M.L., Azar D., Buch J., Ciolino J.B., Naroo S.A., Pall B., Romond K. (2021). CLEAR—Contact lens technologies of the future. Cont. Lens Anterior Eye.

[23] Shioya S., Higashide T., Tsuchiya S., Shimon Z.S., Varidel T., Cerboni S., Mansouri K., Sugiyama K. (2020). Using 24-hr ocular dimensional profile recorded with a sensing contact lens to identify primary open-angle glaucoma patients with intraocular pressure constantly below the diagnostic threshold. Acta Ophthalmol..

[24] Wang Z., Li X., Zhang X., Sheng R., Lim Q., Song W., Hao L. (2021). Novel contact lenses embedded with drug-loaded zwitterionic nanogels for extended ophthalmic drug delivery. Nanomaterials.

[25] Kumar N., Aggarwal R., Chauhan M.K. (2020). Extended Levobunolol Release from Eudragit Nanoparticle-Laden Contact Lenses for Glaucoma Therapy. Futur. J. Pharm. Sci..

[26] Scientific Committee on Consumer Safety OPINION on Carbon Black (Nano-Form) Association, 15 December 2015, Opinion of the Scientific Committee on Consumer Safety on 2,6-Diaminopyridine [Internet]. https://ec.europa.eu/health/scientific_committees/consumer_safety/docs/sccs_o_144.pdf.

[27] Dixon P., Chauhan A. (2019). Carbon black tinted contact lenses for reduction of photophobia in cystinosis patients. Curr. Eye Res..

[28] Kodama A., Tsukiyama J., Miyamoto Y., Sugioka K., Fukuda M., Shimomura Y. (2016). Effects of colorants on the oxygen transmissibility of colored contact lenses. J. Jpn. CL Soc..

[29] Boonstra B.B. (1967). Mixing of carbon black and polymer: Interaction and reinforcement. J. Appl. Polym. Sci..

[30] Kuwahara T., Ito S. (1972). Studies on pigment dispersion in water. II. Shikizai.

[31] Borah D., Satokawa S., Kato S., Kojima T. (2008). Characterization of chemically modified carbon black for sorption application. Appl. Surf. Sci..

[32] Okoye C.O., Zhu M., Jones I., Zhang J., Zhang Z., Zhang D. (2022). An investigation into the preparation of carbon black by partial oxidation of spent tyre pyrolysis oil. Waste Manag..

[33] Kaneko K., Otsuka H. (2020). New IUPAC recommendation and characterization of nanoporous materials with physical adsorption. Acc. Mater. Surf. Res..

[34] Li P., Wang Y., Peng Z., She F., Kong L. (2011). Development of chitosan nanoparticles as drug delivery systems for 5-fluorouracil and leucovorin blends. Carbohydr. Polym..

[35] Parisi E., Garcia A.M., Marson D., Posocco P., Marchesan S. (2019). Supramolecular tripeptide hydrogel assembly with 5-fluorouracil. Gels.

[36] Kuriki R., Hata T., Nakayama K., Ito Y., Misawa K., Ito S., Tatematsu M., Kaneda N. (2019). Tegafur and 5-fluorouracil levels in tears and changes in tear volume in long-term users of the oral anticancer drug S-1. Nagoya J. Med. Sci..

[37] Ueda H., Nagai T. (1978). Adsorption of sulphonylureas by carbon black from aqueous solution. Chem. Pharm. Bull..

[38] (2024). Sterilization of health care products — Moist heat — Part 1: Requirements for the development, validation and routine control of a sterilization process for medical devices.

[39] Nakamoto M., Sato K., Yamauchi T., Morisaki S., Tonogi M., Yamane G., Tanaka Y., Ichiba H., Inoue Y. (2009). Serum concentrations of FT and 5-FU after S-1 oral or tube-administration for chemotherapy in oral cancer patients. (oral administration, a simple suspension method). Shikwa Gakuho.

