# Supplementary material for: Synthesis and Evaluation of Carbon Black-Containing Hydrogels for the Adsorption of 5-Fluorouracil
Source: Gels. 2025 Nov 18;11(11):919. doi: 10.3390/gels11110919 (PMC12652628; doi:10.3390/gels11110919)
Supplement: Supplementary file 1 [file gels-11-00919-s001.zip › gels-3971145-supplementary.pdf]

Supplementary material

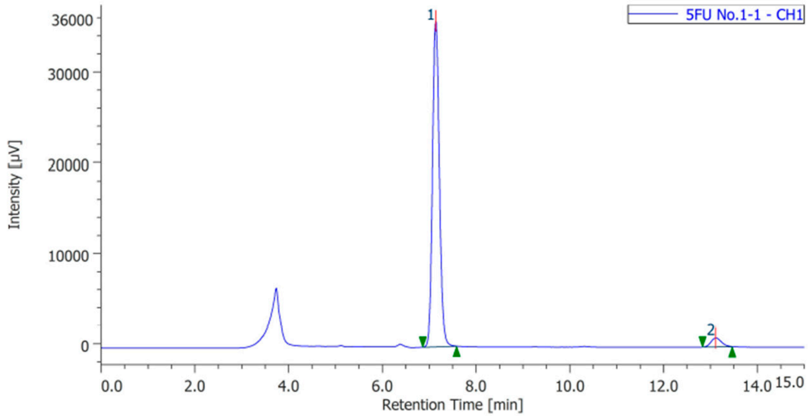

Figure S1. High-performance liquid chromatography peaks of CB1

A 5-FU peak was observed at 7.1 min, a solvent and injection peak at 3.8 min, and a CB-derived peak at 13.1 min, confirming separation. Similar HPLC chromatograms were obtained for all CBs. A calibration curve was prepared using 5-FU standard samples, and the concentration was quantified from the peak area of 5-FU.

5-FU, 5-fluorouracil; CB, carbon black; HPLC, high-performance liquid chromatography
